# Influencing factors of health screening among retirees: an extended TPB approach

**DOI:** 10.3389/fpubh.2024.1320920

**Published:** 2024-03-22

**Authors:** Jiayi Xu, Younghwan Pan, Qiongzhen Li

**Affiliations:** ^1^Department of Smart Experience Design, Graduate School of Techno Design, Kookmin University, Seoul, Republic of Korea; ^2^Shizong County People's Hospital, Yunnan, China

**Keywords:** health screening, the theory of planned behavior, self-efficacy, participate behavior, retirement

## Abstract

**Background:**

Health screenings are promoted worldwide as they help detect and prevent overall health issues. Despite expanding coverage, the participation rate among the retired population has not significantly increased. Given the special role of health screenings in promoting health and healthy aging, understanding the behavioral intentions, and influencing factors of retirees’ voluntary participation in health screenings is crucial. This study aims to explore the participation intentions in health screenings among the Chinese retired population by integrating the Theory of Planned Behavior (TPB) and Self-Efficacy (SE).

**Methods:**

This study used a cross-sectional design to conduct an online questionnaire among 311 retirees in 2023. The questionnaire, tailored for the Chinese retired population, combines the TPB theory and Self-Efficacy theory, including demographic structure, the basic structure of TPB, and SE.

**Results:**

A Structural Equation Modeling (SEM) approach was used to identify factors related to health screening behaviors. Of the respondents, 311 completed the survey (88.9% response rate). The most crucial determinant of health examination behavior was behavioral intention, with a correlation score of (1.524, *p* < 0.001). Significant correlates of behavioral intention included Subjective Norms (SN) and Self-Efficacy (SE), followed by Perceived Behavioral Control (PBC) and Attitude (AT), with correlation scores of (0.401, *p* < 0.001), (0.339, *p* < 0.001), (0.082, *p* < 0.001), and (0.060, *p* < 0.05), respectively.

**Conclusion:**

This study provides insights for enhancing the willingness and behavior of retirees to participate in health screenings.

## 1 Introduction

Health screening helps to detect and prevent the overall health of individuals, and maintaining good physical health can improve the quality of life of individuals and enhance psychological and social well-being (1). Health screening refers to the practice of checking disease indicators before symptoms appear. A considerable proportion of health screening in China are offered as packages—often including X-rays, ultrasound, CT, and an extensive array of tumor markers and genetic tests (2). In China, although health screening coverage has expanded over the past decade, most potential beneficiaries remain unscreened and underserved (3, 4). Population aging is the new normal for society in this century (5). With the continuous development of the social economy, retirees’ health and well-being are affected by income, free time, social roles, and life partners of life (6–8). Given the unique role that health screening plays in promoting health and healthy aging, it is crucial to understand the behavioral intentions and factors influencing voluntary participation in health screening among retirees. Previous research has explored the importance of Health screening from different perspectives, including community health centers, urban populations, rural populations, lifestyle interventions (9–12), and disease screening (13–15). Many retirees were not fully aware of the benefits of health screening to themselves and their families. They believed that there was a low risk of acquiring underlying diseases and getting sick, so they did not need to attend health screening. A lack of awareness of health screening was a major reason why people did not participate.

The Theory of Planned Behavior (TPB) theoretical framework is often used to explain behavioral intentions and has been widely applied to health behavior (16). Its essence is a social cognitive theory of decision-making processes, considered an effective theoretical framework for behavioral guidance. In the context of health behavior, retiree participation in Health screening behavior (PB) is essentially determined by participation intention (PI); among them, PI is influenced by the attitude of whether to participate in health screening (AT), the opinions of people around retiree about health screening (SN), and the individual’s decision to participate in health screening (PBC). The theoretical framework provides a promising avenue for understanding retirement groups’ behavioral intentions and behaviors in Health screening. A large number of studies (17–19) proved that TPB can successfully predict health behaviors and found that TPB can effectively predict different types of Health screening behavior (20, 21). Therefore, the purpose of this study was to investigate the psychological factors related to health screening intentions among Chinese retirees and to understand retirees’ participation intentions and motivations, which could help improve the examination rate. We hypothesized the applicability of TPB in explaining the role of Chinese retirees participating in health screening.

Along with the TPB model, many studies have combined SE for discussion; SE is another important decision-making factor that predicts health behavior (22–24). Researchers have found that self-efficacy positively impacts one’s intention to engage in activities and effectively increases the likelihood of that behavior. It is a strong health predictor as it affects patients’ certainty about their ability to perform recommended behaviors to improve their health. These findings have been demonstrated in various area studies relating to health behavior (25–27). The contributions of this study are as follows.

Previous research mainly focused on health screening in the clinical field or big data screening in public health. This study predicts the participation intention of current health screening and the influencing factors of participation behavior from the perspective of personal psychology and behavior. Then, this study adds SE to the TPB theoretical framework and verifies the feasibility of the TPB extended model in the health screening behavior of retired people. Finally, this study demonstrates the inherent mechanism between the four prediction dimensions. Through the analysis of 311 valid questionnaires, it explores the influencing mechanism of retirees’ willingness to participate in physical examinations, aiming to help decision-makers formulate relevant policies and regulations and provide Practitioners provide reliable project design guidance. And more clearly identify participants’ participation intentions and participation status from a practical perspective.

The remainder of this paper is organized as follows. Section 2 examines the literature on the TPB and SE and explains the theoretical background. Additionally, we propose several hypotheses. In sections 3 and 4, we describe our data and present the descriptive statistics. Section 5 analyzes the research results and the limitations and suggestions. Finally, Section 6 provides a conclusion highlighting future research implications.

## 2 Literature review

### 2.1 Theory of planned behavior

The theoretical framework of this study was adapted from the TPB. They propose that an individual’s intention to engage in a specific behavior is the strongest predictor of that behavior (16). The relationships between these factors (AT, SN, and PBC) in the TPB model all have been supported when applied to different health behaviors. Such as McEachan’s review of the literature discussing health-related behaviors surveyed 206 papers using the TPB theory, and the authors concluded that TPB was a good predictor of health behaviors (28). Moreover, the TPB has also been used as a theoretical framework for designing health behavior interventions, including promoting physical activity (29) or protect oral health (30). They expand the explanatory power of TPB.

In addition, based on the TPB model, most studies of participation in Health screening intention or behavior focus on cancer screening for men or women. For example, one study found women’s promotion of cervical cancer screening rates (31), Sun’s studies in rural women’s willingness to participate in breast cancer (32), and Sieverding’s research men—participation in prostate cancer screening (33). The authors found that TPB use and Health screening in retirees have not been studied. Therefore, this research aims to explore the feasibility of using the TPB theory to investigate the participation of retired individuals in Health screening in China.

#### 2.1.1 Attitude toward the participate intention

Attitude (AT) reflects an individual’s evaluation of performing health screening, seen as either positive or negative (34, 35). This concept suggests that attitudes toward specific health behaviors, such as cancer screenings, significantly influence participation, 2023; Sun et al. (31, 32). Research in retired populations indicates that attitudes can predict engagement in activities like sports (36) and influence intentions to seek mental health help (37). A study on older adults with hearing loss highlighted that those positive attitudes toward their condition encouraged health-seeking behaviors (38). Thus, AT is thought to involve intentions to undergo health screenings, associated concerns or emotions, and the belief in the health benefits of health screenings.

#### 2.1.2 Subjective norm toward the participation intention

Subjective norms (SN), reflecting perceived social pressure, significantly influence health behavior intentions. Studies indicate SN’s strong impact on specific health actions, with norms categorized into descriptive (observing others’ actions) and injunctive (beliefs about what significant others think one should do) (39–41). Yap found that higher perceived social pressure increases the intention to participate in health screenings (42). Similarly, recommendations significantly affect decisions like HPV vaccination among young women (43) and seeking cancer help among retired men (44). Chang’s study highlighted the media’s role in shaping behaviors like hearing aid use among the older adult (45). In our study, retirees’ health checkup decisions were influenced by significant others, with family, friends, and authority figures playing crucial roles in their choices. Online information also impacts their perceptions and decisions regarding health screenings.

#### 2.1.3 Perceived behavioral control toward the participation intention

Perceived behavioral control (PBC) reflects an individual’s belief in their capability and control over actions, influencing the ease of performing behaviors (34). Recognized as a critical health asset, PBC correlates with health behavior intentions and actions (46, 47). Studies show PBC linked to behaviors like responsible drinking (48) and parental actions to prevent children’s myopia (49). In type 2 diabetes management, PBC significantly influences treatment participation, with patients more willing to undergo injections believing in better disease control (20, p. 2). Others highlighted PBC’s role in mitigating aging-related declines (50) and in promoting physical activity among older adults (32, 51). This study posits that retirees’ PBC affects their decision and ability to engage in health screenings.

#### 2.1.4 Participation intention and behavior in health checks

Understanding the role of intention in participating in health behaviors is critical, as it directly affects participation in health Screening. Intention has been proven to be a direct determinant of behavioral orientation in the mental health management of the older adult (52), diabetic patient care (53, 54), and oral health (55). Literature on CCS health screenings indicates intentions can account for behavioral differences (56). However, intentions do not always result in actions, as seen in a study on older adults with osteoarthritis in Portugal, where intention had little effect on physical activity (57). The participation intention we discuss in this study is the degree of readiness to perform health screening behaviors, which can include positive and negative attitudes and is influenced by the advice of significant others.

### 2.2 Self-efficacy toward the participation intention

Self-efficacy, reflecting confidence in managing health outcomes and perceived health competence, strongly predicts health behaviors (58, 59). It is linked to behaviors that improve older adult health (60, 61)and influences life satisfaction (62), community health activity engagement (63), and health management (64, 65). Schwarzer highlighted that action self-efficacy predicts preventive health actions in the older adult (66). Retirement impacts seniors’ health behaviors, with high self-efficacy post-retirement enhancing health actions, such as increased smoking cessation rates among retirees (67, 68). Leisure self-efficacy also improves retired individuals’ quality of life (69). Despite these findings, the causal link between self-efficacy and behavior requires further exploration (70). This study examines self-efficacy as a predictor of retirees’ engagement in preventive health measures, specifically in health screening participation, including planning and adherence to health screening activities.

### 2.3 Development of hypotheses

This research examines the influence of retirees’ internal determinants on decision-making and participation in health screening activities. We put forward two research questions:

RQ1: What specific reasons influence retirees’ voluntary participation in Health screening?
RQ2: To what extent do these factors influence the likelihood of retirees voluntarily engaging in health screening behaviors?

These two research questions aim to identify factors contributing to voluntary participation in health screening among retired individuals. Additionally, they seek to determine how these factors impact retirees’ intentions to participate in Health screening voluntarily. These research questions are essential as they assist practitioners or policymakers in better serving retired individuals and addressing the challenges associated with the aging population.

Based on the TBP model and prior studies, the following hypothesis was proposed:

*H1*: The AT of retirees toward health screening positively correlates with their willingness to participate in Health screening.

*H2*: The SN of retirees regarding health screening positively correlates with their willingness to participate in Health screening.

*H3*: Retirees' PBC to participate in health screening and intention to participate are positively related.

*H4*: Retirees' SE to participate in health screening and intention to participate are positively related.

*H5*: Retirees' PI in Health screening and the PB of the retirees are positively correlated.

**Figure 1:**
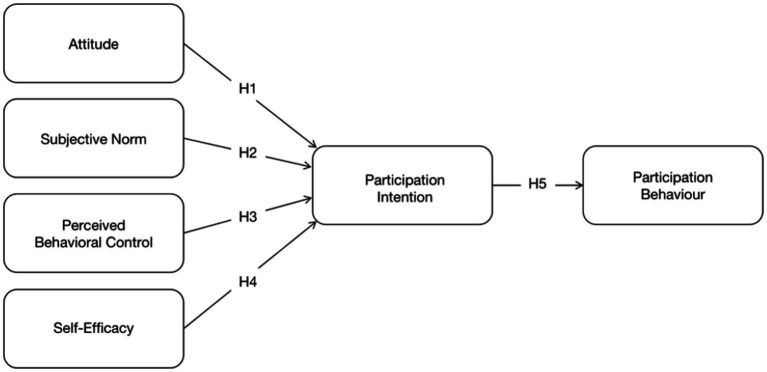
Participation behavior model based on TPB.

Based on the above hypotheses, we could add new variables (SE) to the TPB model on a reasonable basis. The research examines survey data on the retired population’s intentions to participate in Health screening and will relate influencing factors. Combined with the existing literature, the research hypotheses and the model adopted in this study were developed based on TPB, shown in Figure 1.

## 3 Methods

### 3.1 Instrument

To test the above hypothesis (Figure 1), this study used multi-item scales to measure each variable, which provides better stability and minimizes measurement error. These well-established scales have been rigorously tested in numerous studies and have shown good reliability and validity. The measurement items of this study were evaluated and refined based on the previous research scale to ensure the validity and reliability of the content and to be more suitable for retirees’ health screening behavior.

The TPB Measures include TPB structure and SE factor (Items are displayed in Table 1). The TPB structure is adapted from the TPB scale (71), and has been used in four previous research studies, Shumeli (72), Xin (31), and Dilekler (73), and Babazadeh (39): AT (Q6-Q9) refers to retirees’ positive or negative emotional feedback on participating in health screening behavior, SN (Q10-Q13) refers to retirees are more likely to be influenced by other influential people when participating in health screening. PBC (Q14-Q17) refers to how retirees can decide how easy it is to participate in health screening. PI (Q22-Q25) refers to the direct motivation of retirees to participate in health screening. PB (Q26-Q29) refers to retirees’ actual behavior of participating in health screening. In addition to the TPB construct, the questionnaire also included SE (Q18-Q21) as antecedents, which was adapted from the Measuring optimistic self-beliefs: A Chinese adaptation of the General Self-Efficacy Scale (75), Stout (74) and Dolatabadi (25), referring to the degree to which retirees feel confident in their ability to manage their health effectively. The measurement items consist of a total of 24 items, and the constructs are measured using a five-point Likert scale, ranging from 1 (representing “strongly agree”) to 5 (defining “strongly disagree”). Likert scales assume that attitudes can be assessed, and that the degree of attitude lies on a linear continuum between strongly agree and strongly disagree. All items used in the questionnaire were translated into Chinese.

**Table 1:** Details of the employed TPB questionnaire.

Variables	Number of items	Sample of items	Sources reference
Attitude (AT)	4	The health screening will help me improve my health if I choose to participate in it.	(71, 72)
Subjective norm (SN)	4	When friends and I talk about health screening, it can make me want to go for one.	(31)
Perceived behavior control (PBC)	4	I can afford to pay for the health screening, and I will go for it.	(39, 73)
Participation intention (PI)	4	I participate in health examination in order to obtain a health report.	
Participation behavior (PB)	4	I have completed the health screening within the planned time.	
Self-efficacy (SE)	4	I am capable of staying healthy even if no one asks me to take a health examination, I do.	(25, 74, 75)

The constructs are measured using a five-point Likert scale, ranging from 1 (representing “strongly agree”) to 5 (defining “strongly disagree”).

To ensure the questionnaire’s content validity, we consulted a panel of experts, including three senior medical professionals specializing in health screening, two university lecturers with expertise in English, and two health educators. Their feedback was instrumental in finalizing the survey questionnaire.

### 3.2 Data collection

To meet the requirements of our study design, considering that we have 29 variables and following the principle that each variable requires at least 10 samples, we needed a minimum of 290 participants. To test the research hypotheses, we collected data from retired people in Shizong County, Yunnan Province. All data were collected between May and July 2023. An online questionnaire was generated on the platform with a unique web link. We used a screening question: “Have you reached retirement age or are preparing to retire? Are you willing to participate in this survey?” to exclude participants who do not meet the age standards and are unwilling to participate in the study to ensure that participants in the questionnaire meet China’s retirement age Conditions include early retirement (45 years for women and 50 years for men) and mandatory retirement (50 years for women and 60 years for men) (76). Through these procedures, a total of 350 questionnaires were distributed. After excluding invalid questionnaires with basically the same answers, logical errors, or response times of less than 15 s, 311 valid responses were obtained, and the overall effective questionnaire response rate was 88.9%. This study complied with the recommendations of the Declaration of Helsinki and was approved by the Institutional Review Board (IRB) of Shizong County, Yunnan Province.

### 3.3 Ethical considerations

This study complied with the recommendations of the Declaration of Helsinki and was approved by the Institutional Review Board (IRB) of Shizong County, Yunnan Province, ethics code/number: YNSZ-IRB-020-20230524.

### 3.4 Sample characteristics

As shown in Table 2, in the demographic analysis, the age group of 50–64 years had the highest number of participants in Health Screening, accounting for 47.91% of the total. The age group of 65–74 years accounted for 36.66%. Regarding gender distribution, females accounted for 45.02%, while males accounted for 54.98%. Regarding education, 45.98% had a high school education or below, 33.44% had a vocational education, 13.50% had a bachelor’s degree, and 7.07% had a graduate degree or above. Regarding the participants’ occupations before retirement, the highest proportion was observed among employees of state-owned enterprises and public institutions, accounting for 52.41%. Regarding monthly income distribution, 45.34% had incomes between 2001 and 5,000 yuan, while 42.77% had revenues ≤2000 yuan.

**Table 2:** Sample characteristics (*N* = 311).

Variable	Categories	Frequency	*N* (%)
Age	45–49	18	5.79
	50–64	149	47.91
	65–74	114	36.66
	Above 75	30	9.65
Gender	Male	171	54.98
	Female	140	45.02
Education	Up to secondary school	143	45.98
	College	104	33.44
	Undergraduate	42	13.50
	Graduate or higher	22	7.07
Occupation	Civil servants	62	19.94
	Employees of state-owned enterprises or public institutions	163	52.41
	Self-employed	60	19.29
	Unemployed	26	8.36
Monthly income	Under 2,000	133	42.77
	2,001–5,000	141	45.34
	5,001–10,000	34	10.93
	Above 10,001	3	0.96

### 3.5 Data analysis methods

This research employed structural equation modeling (SEM) to test the conceptual framework. We used SPSS 26.0 and AMOS 26.0 to analyze the main factors influencing retirees’ participation in health examinations. Firstly, frequency analysis was conducted to determine the participants’ general characteristics. Secondly, confirmatory factor analysis was performed using the AMOS 26.0 software to validate the dimensions and validity of the variable factor structure.

## 4 Results

### 4.1 Measurement tool assessment

#### 4.1.1 Results of the reliability and validity test

This section uses confirmatory factor analysis (CFA) to examine the agreement between the five factors and the theoretical model. Total reliability (CR) and average variance extraction (AVE) evaluated the indicators’ convergent and discriminant validity. AVE values >0.5 and CR values >0.7 are generally recommended (77). In this research, Cronbach’s alpha values of all variables are above 0.7, indicating good internal consistency reliability. All variables’ composite reliability (CR) values exceeded 0.7, indicating sufficient convergent validity (78). In addition, the AVE values of the six factors involved in this research (AT et al.) were all greater than 0.5, indicating that the scale data have excellent discriminant validity (Table 3). The results show that the scale has good reliability and validity.

**Table 3:** The results of construct assessment.

	Mean	SD	SFL	CA	CR	AVE
AT				**0.853**	**0.901**	**0.694**
Q6 (AT1)	3.73	1.141	0.839			
Q7 (AT2)	3.81	1.095	0.82			
Q8 (AT3)	3.78	1.109	0.852			
Q9 (AT4)	3.92	1.087	0.821			
SN				**0.801**	**0.87**	**0.627**
Q10 (SN1)	3.84	1.074	0.782			
Q11 (SN2)	3.89	1.037	0.81			
Q12 (SN3)	3.89	1.022	0.792			
Q13 (SN4)	4.01	1.011	0.782			
PBC				**0.864**	**0.907**	**0.71**
Q14 (PBC1)	3.66	1.147	0.847			
Q15 (PBC2)	3.68	1.164	0.852			
Q16 (PBC3)	3.7	1.132	0.838			
Q17 (PBC4)	3.76	1.1	0.834			
SE				**0.818**	**0.879**	**0.645**
Q18 (SE1)	3.86	1.096	0.84			
Q19 (SE2)	3.91	1.017	0.775			
Q20 (SE3)	3.91	1.032	0.815			
Q21 (SE4)	4.03	0.967	0.782			
PI				**0.795**	**0.867**	**0.619**
Q22 (PI1)	3.66	1.124	0.798			
Q23 (PI2)	3.75	1.101	0.789			
Q24 (PI3)	3.71	1.089	0.777			
Q25 (PI4)	3.85	1.044	0.783			
PB				**0.812**	**0.877**	**0.64**
Q26 (PB1)	3.94	1.019	0.806			
Q27 (PB2)	3.93	1.003	0.791			
Q28 (PB3)	3.96	1.006	0.782			
Q29 (PB4)	4.04	1.028	0.82			

AT, Attitude; SN, Subject norm; PBC, Perceived behavioral control; SE, Self-efficacy; PI, Participate intention; PB, Participate behavior; SD, Standard deviation; SFL, Standardized factor loading; CR, Construct reliability; CA, Cronbach’s alpha; and AVE, Average variance extracted.

#### 4.1.2 Results of the reliability and validity test

Table 4 shows the results of the discriminant validity test. The square root values of the AVEs for all facets were higher than the inter-facet correlations, demonstrating sufficient discriminant power.

**Table 4:** Discriminant validity test.

	AT	SN	PBC	SE	PI	PB
AT	**0.833**					
SN	0.292	**0.792**				
PBC	0.389	0.339	**0.843**			
SE	0.335	0.319	0.331	**0.803**		
PI	0.192	0.248	0.214	0.261	**0.787**	
PB	0.387	0.493	0.425	0.507	0.439	**0.800**

AT, Attitude; SN, Subject norm; PBC, Perceived behavioral control; SE, Self-efficacy; PI, Participate intention; PB, Participate behavior; Figures on the diagonal line (in bold) are the square root of the average variance extracted (AVE). Off-diagonal figures show inter-construct correlations.

### 4.2 Assessment of the structural model and the hypotheses

#### 4.2.1 Model fitting

In the first step of hypothesis testing, the structural model was evaluated. Our model shows a good fit for the data. The structural equation fit of the model was (χ^2^ = 871.9, df = 2,449, *p* = 0.00, CMIN/df = 3.502, NFI = 0.739, CFI = 0.797 IFI = 0.799, and RMSEA = 0.090). All fit index values were acceptable according to the established fit criteria (79).

#### 4.2.2 Hypothesis testing and path size significance

**Figure 2:**
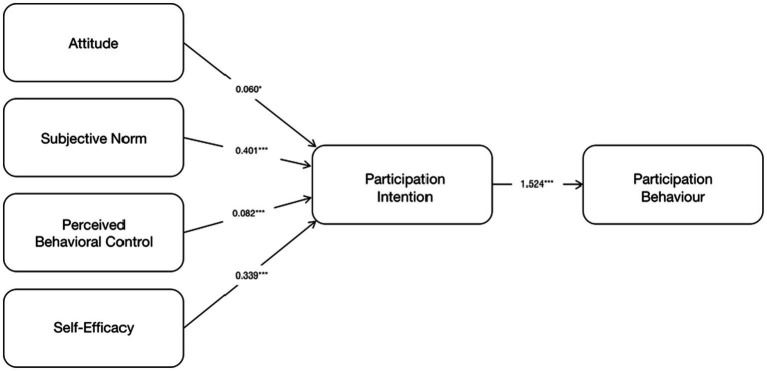
Standardized structural estimates and hypotheses tests, ^*^p < 0.05, ^***^p < 0.001.

This study calculated the path coefficient and *p* value through bootstrapping with a sample of 311 subjects. As shown in Table 4; Figure 2, all hypotheses are supported at a significant level of *p* < 0.05 or *p* < 0.001.

In this research, 311 retirees were surveyed, and the path coefficient and *p* value were calculated. As shown in Table 5; Figure 2, the intention-oriented hypothesis of participating in the health examination shows that the relationship between AT and PI is statistically significant when *p* < 0.05, showing a positive effect (0.060), which supports hypothesis H1. Secondly, the relationship between the three psychological factors (SN et al.) and the intention to participate in the behavior is statistically significant when *p* < 0.001, SN and SE have a significant effect on the PI had a significant positive effect (0.401 and 0.339). PBC had a positive correlation (0.082) with PI, supporting hypotheses H2, H3, and H4, respectively. Finally, PI has a significant positive correlation (1.524) to PB, so hypothesis H5 holds.

**Table 5:** Hypothesis testing and path size significance.

Hypothesis/Path	Estimate	S.E.	C.R.	*t* value	Results
Hypothesis 1. AT→PI	0.060^*^	0.023	2.576	0.981	Supported
Hypothesis 2. SN→PI	0.401^***^	0.079	5.054	2.513	Supported
Hypothesis 3. PBC→PI	0.082^***^	0.024	3.357	1.385	Supported
Hypothesis 4. SE→PI	0.339^***^	0.065	5.240	2.751	Supported
Hypothesis 5. PI→PB	1.524^***^	0.287	5.309	8.591	Supported

AT, Attitude; SN, Subject norm; PBC, Perceived behavioral control; SE, Self-efficacy; PI, Participate intention; and PB, Participate behavior; ^*^p < 0.05, ^***^p < 0.001.

## 5 Discussion

This study investigated the relationship between retirees’ intention to participate in health screening and actual participation by extending the TPB and incorporating SE. Research shows that the behavior of retirees to participate in health screening depends on their intentions and is directly affected by SN and SE. SN and SE significantly affect retirees’ participation behavior, with participation intention as a mediator. PBC and AT have weak effects on retirees’ intention to participate. All hypotheses in the model were supported, confirming that TPB is an acceptable theoretical basis for this study. This finding is consistent with previous TPB-based studies conducted in the United States (80), China (81, 82), and Europe (57).

This chapter discusses the theoretical and practical implications of our study in detail. Finally, we will also address the limitations of our study and provide recommendations for future research in this area.

### 5.1 Predictive factors

#### 5.1.1 Predictive factors of intention for health screening

The results indicate that SN, SE, PBC, and AT positively impact retirees’ intention to participate in health Screening.

The results found that SN is the most critical predictor of retirees’ behavioral intention to participate in health check-ups, and it has the highest correlation with PI. This result echoes previous research that SN can well predict intentions in health behaviors (28) from the three aspects of social support, family support, and collectivism (31, 41, 54). It was also found that the external environment positively impacts SN (83), that is, the widespread use of social software and the accessibility of health information in the Internet environment. Social support in social networks is a significant health resource. High levels of social support are associated with better health behaviors and a greater intent to participate in health screening. This is further emphasized by the recent Chollou study (84).

This study confirms the feasibility of incorporating Self-Efficacy (SE) into the Theory of Planned Behavior (TPB), aligning with the views of Fishbein and Cappella that SE can better explain individual behavior within TPB (85). For instance, (86) found that blood donors with higher SE are more positively influenced by their participation experience. Similarly, Oikarinen noted in their study on the dietary habits of obese individuals that those with lower SE face more challenges in weight control (26), highlighting the importance of SE in self-care behaviors (87). In summary, within the TPB framework, researchers explain that individuals with higher perceived social acceptance and stronger SE are more likely to engage in certain behaviors in reality (88), a viewpoint our research also supports. Furthermore, we found a positive link between Social Norms (SN) and SE, where an increase in SN is accompanied by a rise in SE, similar to our findings, anothers also showed thereby positively affecting the intention to participate in health behaviors (89). In health behavior promotion, the external environment’s impact on SE could either facilitate or hinder self-care behaviors (90).

This study also discusses the role of SE in TPB, which is consistent with the conclusion of many researchers that SE better explains individual behavior in TPB (85). For example, in studies on voluntary blood donation behavior, experienced blood donors with higher SE were more susceptible to the influence of their participation behavior (86). In a study of poor eating habits in obese individuals, those with lower SE were less able to control their weight, making successful weight loss more challenging (26). Overall, within the framework of the TPB, the researchers explained that individuals with higher perceived social acceptance and more robust SE are more likely to perform certain behaviors in reality (88), and our research supports this view. In addition, SN and SE influence each other. When SN increases, SE will also increase. SE can increase an individual’s subjective commitment to perform a specific health behavior and fulfill others’ expectations, thus positively affecting the intention to participate (89).

In addition, there is a positive correlation between PBC and PI. However, the strength of this relationship is weaker compared to SN and SE, which is consistent with previous studies in the literature (91, 92). Our findings support this notion. When SE and PBC coexist, SE can serve as a better measure (93). In 2020, Ajzen proposed after further research on TPB that both refer to people’s belief in their ability to perform a given behavior. However, operationally, PBC and SE are often assessed different ways (94). This also confirms previous research results.

Interestingly, a significant difference found in this study compared to previous studies is that AT’s impact is smaller than other influencing factors. Our survey on AT is consistent with a small number of prior investigations (31, 54). There may be several reasons for this result. First, the retired group has a low level of education and limited knowledge of health examinations, resulting in distrust and disparity in health. This is consistent with the research of Zheng (95) and Guo (96). When patients have a low disease background, health information avoidance will make people face the disease or take preventive measures. Based on these factors, individuals may exhibit avoidance attitudes (AT) toward health examinations, which does not positively affect their willingness to participate (PI). Secondly, another possible reason for this result may be the existence of a neutral attitude (97, 98). Recent research (99) confirms that an apathetic attitude is negatively related to behavior. In this study, retirees showed no apparent positive or negative attitudes toward health screenings, defined as neutral attitudes. Another possible explanation for speculation is that attitudes toward current health research differ due to different target types (health protection and health risks) (40). Attitudes show great predictive power when directed toward high-reward health behaviors—health-risk behaviors, such as smoking or alcohol abuse. Therefore, the impact of AT on PI is relatively small in this case. Since this study is an empirical study, local retirees may be neutral in their attitudes toward whether to participate in health screening, and there may be other unmeasured antecedent variables that impact AT results. The measurement of AT and its role in health screening PI warrants further study.

#### 5.1.2 Predictive factors of health screening behavior

In this research, we also determined the influence coefficient of PI-PB, the final link of TPB, which further confirmed that TPB is suitable for studying voluntary behavior and its applicability in health behavior. Accumulated evidence shows that intention is the most crucial factor in the theory of planned behavior. Best predictor. PI effectively promotes the generation of PB in health behaviors, which is consistent with previous findings in the intention-behavior relationship that reasonable intentions may provide better behavior prediction (100–102). This provides strong evidence for the decisive impact of PI on PB. In our study, the effects of AT, SN, PBC, and SE on participation intention were all valid, and the impact of PI on retiree health checkup PB was not inconsistent with previous studies. This provides strong evidence for the decisive impact of PI on PB. Retirees have a strong willingness to participate in health screening. In previous studies (67), people were restricted from participating in various health examinations, which may be why people are not enthusiastic about participating in health examinations. Because of intention-behavior congruence, this also means that people are less likely to engage in healthy behaviors when intentions are not strong.

This study aims to identify the factors that motivate Chinese retirees to participate in health screenings and how they influence their participation intentions by incorporating Self-Efficacy (SE) into the Theory of Planned Behavior (TPB). A distinctive aspect of this research is the addition of new variables to overcome the TPB model’s limitations in explanatory power. This approach coincides with Ranjbaran’s study, where SE not only enhances the TPB’s explanatory power for health participation behavior but also provides a unique perspective for discussions on health promotion and public health (103). Enhanced the explanatory power of the model in health participation behavior. This study focuses on exploring the individual participation behavioral intentions of retired people through the perspective of TPB. This unique perspective contributes to discussing health promotion, public health, and active aging. Third, to the best of the researchers’ knowledge, this study is one of the few empirical studies on retiree health screening behavior and intention to participate. The results of SEM show that all hypotheses in the extended TPB model are significantly supported, and the final data are consistent with the theoretical predictions. This makes this study significant and further proves TPB’s predictive utility. Judging from the results of this study, it is valuable for us to extend the TPB model, which will validate its effectiveness in health promotion research. To explore the effective expansion of the TPB expansion model in health participation behavior in public health and healthcare.

### 5.2 Implications

#### 5.2.1 Theoretical implications

This study provides theoretical and practical research. The theoretical contributions derived from this study are, first, an in-depth exploration of the particularities of the retired group, considering that their life situations, personal needs, and experiences are significantly different from those of other age groups. Therefore, their socio-psychological factors, including AT, SN, PBC, and SE also show different characteristics. Notably, unlike previous research, we found that PBC did not predict behavioral intentions among retired people as strongly as expected. We also observed differences in people’s AT for health checks in different social backgrounds. SN of retired groups and SE of new entrants are significant predictors. Therefore, an in-depth understanding of these factors is crucial to improving the intention of retired people to participate in health checks. Secondly, the study introduced self-efficacy and incorporated it into the TPB model, which can better adapt to the needs of this retired group. The results suggest that targeting participation intentions among retired people may require particular emphasis on subjective norms and self-efficacy rather than just changing attitudes. This theoretical expansion helps to understand better the decision-making process and behavioral intentions of retired people and provides more comprehensive evidence for health promotion and intervention. Finally, by integrating social psychology (SE) and decision-making theory (TPB), we provide a broader perspective on understanding the decision-making process for health behaviors. This integration fills the theoretical gap between the two and helps better meet retired groups’ needs in health behavior intervention. By focusing on this specific group, we help fill the knowledge gap about the health behaviors of retired people. Therefore, it is significant to study the relationship between participation intentions and participation behaviors in retired people. With these in mind, we recommend incorporating research samples into health management studies of older adults to understand better and address their needs.

#### 5.2.2 Practical implications

Practically, research on retirees’ health checkup willingness provides valuable suggestions for improving the follow-up health checkup service process. First, our findings highlight the importance of subjective norms and self-efficacy. Self-efficacy is vital among people aged 25–65, and personal perceptions are highest during this period. In contrast, after age 65, self-efficacy will weaken with subjective perception, and mental and physical health will decline, damaging personal autonomy. However, family support and social support will significantly improve this situation. Healthy psychological and social factors determine the health outcomes of retired people, and social support has profound health benefits for them. As a social and psychological pressure, retirement has a huge impact on retired people. This can improve SE through strategies such as planning a series of health screening.

On the other hand, both interpersonal influence and external influence have a positive impact on subjective norms. Among them, social networks (i.e., social support) under the Internet are the best embodiment of external power. In China, most retirees always stay on their mobile devices, and their daily lives must be separated from the mobile Internet. Social software allows people to communicate more frequently, quickly, and effectively and is more susceptible to their influence. The Internet’s rapid development has reduced the lag in obtaining external information. They can promptly obtain information that is difficult to distinguish between true and false, feedback from mass media, recommendations from merchants, and even advertisements based on geographical location, which make them more susceptible to external influence. Health service operators can comprehensively promote the benefits of health examinations and the importance of health through the Internet and can also apply them to improve lifestyles after health examinations. For example, subjective norms can be reinforced through regular reminders from family, friends, doctors, or health management software, as well as relevant news notifications from government policies and media agencies. These measures will help improve customer satisfaction with health examination services, improve the quality of health examination services, and help formulate relevant policies and plans for health checks services. Since 2018, Chinese physical examination institutions have been increasing year by year and launching physical examination packages for different types of groups. A complete health examination service process can improve customers’ overall health examination experience and willingness to conduct regular health checks.

#### 5.2.3 Policy implications

Finally, this study provides some policy implications. This study discusses factors that significantly influence the participation experience of the retired population. It is worth pointing out that regular health checks have always been a healthy lifestyle recommended by the government, especially for controlling blood pressure and blood sugar, which is crucial for preventing most chronic diseases. The government encourages retired people to use these resources to actively participate in health check-ups by providing free health checks opportunities and publishing and disseminating relevant information. In recent years, innovative medical care and healthy aging have become increasingly popular in China, and more and more hospitals and medical institutions have continued to upgrade and improve the quality of medical services. Policies such as smart medical care and healthy aging are continuously optimized and upgraded in this process. Future policymakers can refer to these related influencing factors when discussing future policies or regulations on intelligent healthcare and healthy aging. In addition, as healthy aging and smart healthcare continue to develop, and more and more institutions invest in them, health examination policies will face many challenges, such as addressing ethical issues, privacy concerns, and individual rights issues when formulating examination activities. Our study takes a first step toward addressing these challenges by identifying specific influencing factors that may contribute to these problems. These influencing factors provide us with valuable entry points to address these challenges.

### 5.3 Limitations and future directions

This research identified three limitations that should be considered when interpreting the results. Firstly, due to the limitation of sample size, our research can only represent some retired individuals, which may affect the generalizability of the findings. Additionally, our model may not include other potential influencing factors, such as the quality of health examination services and economic factors. Including these factors could yield different results and provide a more comprehensive understanding of the factors influencing retired individuals’ intention to participate in health examinations. Lastly, this research is based on a cross-sectional survey with a single dataset, which limits the ability to establish causal relationships. The research did not consider the time frame (e.g., “in the past,” “before retirement,” or “soon to retire”) for investigating the mindset changes before and after retirement. It would be valuable for future research to explore longitudinal changes in the attitudes of retired individuals toward health examinations if the survey data allow for such analysis.

## 6 Conclusion

This study delves into the relationship between retirees’ intentions to participate in health screenings and their actual behavior by extending the TPB and incorporating SE. The findings reveal a significant positive impact of SN and SE on retirees’ intentions to participate in health screenings while also confirming the mediating role of intention between AT, SN, PBC, and SE. These results are consistent with previous TPB-based studies both domestically and internationally, further emphasizing the importance of considering SE in health behavior research. It further proves the predictive utility of TPB and its extended model in health promotion research, offering a new perspective on understanding retirees’ health screening behaviors, especially in assessing how social support and personal beliefs influence health behavior intentions. Additionally, by including SE as a component of the TPB model, this study enhances the model’s explanatory power in explaining health participation behavior, providing a unique perspective for discussions on health promotion, public health, and active aging and laying a theoretical foundation for future health promotion strategies.

On a practical level, this study offers valuable insights for health screening service companies and policymakers. It highlights the need to pay special attention to enhancing retirees’ sense of social norms and SE when designing health screening services and related policies to promote their active participation. Moreover, the study’s findings support the idea that future policies should focus more on retirees’ health screening experiences to increase their willingness and frequency of participation in health screenings.

Despite its theoretical and practical contributions, this study has limitations, such as sample representativeness and consideration of potential influencing factors. Therefore, future research needs to explore other factors that may influence retirees’ health screening behaviors further and how different interventions can effectively increase their willingness to participate.

This study underscores the importance of understanding and enhancing social norms and SE in promoting retirees’ participation in health screenings. By designing interventions targeting these factors, we can more effectively promote health management among retirees, thereby improving public health standards and the quality of life for the older adult.

## Data availability statement

The original contributions presented in the study are included in the article/supplementary material, further inquiries can be directed to the corresponding author.

## Ethics statement

This study complied with the recommendations of the Declaration of Helsinki and was approved by the Institutional Review Board (IRB) of Shizong County, Yunnan Province, ethics code/number: YNSZ-IRB-020-20230524.

## Author contributions

JX: Conceptualization, Formal analysis, Methodology, Software, Supervision, Validation, Writing – original draft. YP: Supervision, Writing – review & editing. QL: Data curation, Investigation, Writing – original draft.
